# 2-(1,3-Benzothia­zol-2-yl)guanidin-2-ium acetate

**DOI:** 10.1107/S160053681104089X

**Published:** 2011-10-12

**Authors:** Peter N. Horton, Simon J. Coles, Shaaban K. Mohamed, Mahmoud A. A. El-Remaily, A. M. Soliman

**Affiliations:** a School of Chemistry, University of Southampton, Highfield, Southampton SO17 1BJ, England; b Chemistry and Environmental Division, Manchester Metropolitan University, England; c Department of Chemistry, Faculty of Science, Sohag University, Egypt

## Abstract

In the title compound, C_8_H_9_N_4_S^−^·C_2_H_3_O_2_^−^, the cation is essentially planar (r.m.s deviation = 0.037 Å) with the guanidine unit bent out of the plane of the fused-ring system by 4.6 (3)°. In the asymmetric unit, the cations and anions are linked into *R*_2_^2^(8) motifs. In the crystal, further N—H⋯O and N—H⋯N hydrogen bonds link the components into a two-dimensional network.

## Related literature

For the crystal structure of the neutral 2-(1,3-benzothia­zol-2-yl)guanidine mol­ecule, see: Mohamed *et al.* (2011 ▶). For hydrogen-bond motifs, see: Bernstein *et al.* (1995 ▶). 
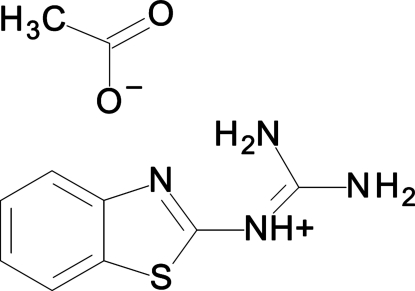


## Experimental

### 

#### Crystal data

C_8_H_9_N_4_S^+^·C_2_H_3_O_2_^−^
*M* *_r_* = 252.30
Orthorhombic, 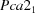

*a* = 12.596 (2) Å
*b* = 11.276 (2) Å
*c* = 8.0936 (12) Å
*V* = 1149.6 (4) Å^3^
*Z* = 4
Mo *K*α radiation
μ = 0.28 mm^−1^
*T* = 120 K
0.14 × 0.10 × 0.02 mm

#### Data collection

Bruker–Nonius APEXII CCD camera on κ-goniostat diffractometer
Absorption correction: multi-scan (*SADABS*; Bruker, 2009 ▶) *T*_min_ = 0.962, *T*_max_ = 0.995
7697 measured reflections
1991 independent reflections
1301 reflections with *I* > 2σ(*I*)
*R*_int_ = 0.116

#### Refinement

*R*[*F* ^2^ > 2σ(*F* ^2^)] = 0.078
*wR*(*F* ^2^) = 0.154
*S* = 1.06
1991 reflections
155 parameters
1 restraint
H-atom parameters constrained
Δρ_max_ = 0.42 e Å^−3^
Δρ_min_ = −0.36 e Å^−3^
Absolute structure: Flack (1983 ▶), 897 Friedel pairs
Flack parameter: 0.3 (2)

### 

Data collection: *COLLECT* (Hooft, 1998 ▶); cell refinement: *DENZO* (Otwinowski & Minor, 1997 ▶) and *COLLECT*; data reduction: *DENZO* and *COLLECT*; program(s) used to solve structure: *SHELXS97* (Sheldrick, 2008 ▶); program(s) used to refine structure: *SHELXL97* (Sheldrick, 2008 ▶); molecular graphics: *ORTEP-3 for Windows* (Farrugia, 1997 ▶); software used to prepare material for publication: *WinGX* (Farrugia, 1999 ▶).

## Supplementary Material

Crystal structure: contains datablock(s) I, global. DOI: 10.1107/S160053681104089X/bx2373sup1.cif

Structure factors: contains datablock(s) I. DOI: 10.1107/S160053681104089X/bx2373Isup2.hkl

Supplementary material file. DOI: 10.1107/S160053681104089X/bx2373Isup3.mol

Supplementary material file. DOI: 10.1107/S160053681104089X/bx2373Isup4.cml

Additional supplementary materials:  crystallographic information; 3D view; checkCIF report

## Figures and Tables

**Table 1:** Hydrogen-bond geometry (Å, °)

*D*—H⋯*A*	*D*—H	H⋯*A*	*D*⋯*A*	*D*—H⋯*A*
N2—H2⋯O12	0.88	1.82	2.671 (8)	162
N3—H3*A*⋯O11^i^	0.88	2.06	2.760 (8)	136
N3—H3*B*⋯N1	0.88	2.05	2.713 (9)	131
N4—H4*A*⋯O12^ii^	0.88	2.03	2.861 (7)	158
N4—H4*B*⋯O11	0.88	1.91	2.790 (8)	173

Symmetry codes: (i) 
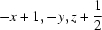
; (ii) 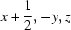.

**Fig. 1.**
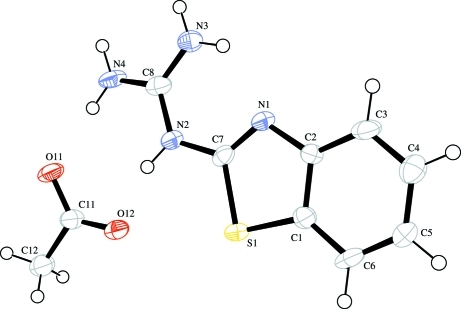
The asymmetric unit of the title compound. Displacement ellipsoids are drawn at the 50% probability level.

**Fig. 2.**
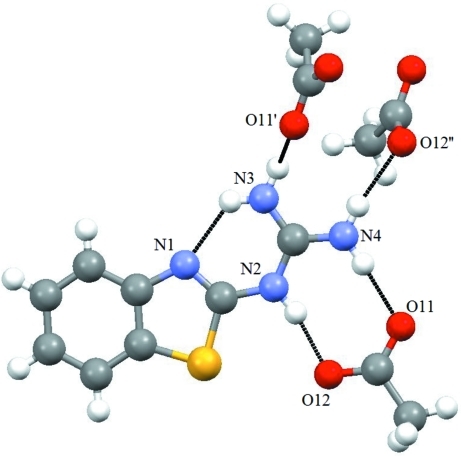
A partial packing diagram for (I), showing the intermolecular and intramolecular hydrogen bonding (symmetry codes: (i) : -x+1, y,z+1/2; (ii) x+1/2,-y,z)

## supplementary crystallographic information

### Comment

The title compound was synthesized and exists as the acetate salt of benzothiazolo-2-guanidinium. The benzothiazolo-2-guanidinium cation is almost planar with the guanidine unit bent out of the plane of the fused-ring system by just 4.6 (3)°. In the asymmetric unit, The cations and anions  are linked into R^2^_2 _(8) motif (Bernstein, *et al.*, 1995). The crystal packing is stabilized by intermolecular hydrogen bonds involving the cations and acetate counter-ions, Table 1, Fig.2.

### Experimental

A mixture of 1 mmol of 2-guanidyl benzothiazole with few drops of glacial acetic acid was heated in ethanol for 2 hours. The mixture was left at room temperature for two days to afford the shiny white crystals of benzothiazolo-2-guanidinium acetate in 94% yield. The single-crystal was obtained from a slow evaporation of the ethanolic solution of product over two days.

### Refinement

H atoms were positioned geometrically [C—H = 0.95 or 0.98 Å and N—H = 0.88 Å] and refined using a riding model, with *U*_iso_(H) = 1.2*U*_eq_(C) or *U*_iso_(H) = 1.5*U*_eq_(C) respectively and *U*_iso_(H) = 1.2*U*_eq_(N).

### Crystal data

**Table:**

C_8_H_9_N_4_S^+^·C_2_H_3_O_2_^−^	*D*_x_ = 1.458 Mg m^−^^3^
*M**_r_* = 252.30	Melting point = 463–465 K
Orthorhombic, *P**c**a*2_1_	Mo *K*α radiation, λ = 0.71073 Å
Hall symbol: P 2c -2ac	Cell parameters from 2099 reflections
*a* = 12.596 (2) Å	θ = 2.9–27.5°
*b* = 11.276 (2) Å	µ = 0.28 mm^−^^1^
*c* = 8.0936 (12) Å	*T* = 120 K
*V* = 1149.6 (4) Å^3^	Plate, colourless
*Z* = 4	0.14 × 0.10 × 0.02 mm
*F*(000) = 528	

### Data collection

**Table:**

Bruker–Nonius APEXII CCD camera on κ-goniostat diffractometer	1991 independent reflections
Radiation source: Bruker–Nonius FR591 rotating anode	1301 reflections with *I* > 2σ(*I*)
10 cm confocal mirrors	*R*_int_ = 0.116
Detector resolution: 4096x4096pixels / 62x62mm pixels mm^-1^	θ_max_ = 25.0°, θ_min_ = 3.2°
φ and ω scans	*h* = −14→14
Absorption correction: multi-scan (*SADABS*; Bruker, 2009)	*k* = −12→13
*T*_min_ = 0.962, *T*_max_ = 0.995	*l* = −9→9
7697 measured reflections	

### Refinement

**Table:**

Refinement on *F*^2^	Secondary atom site location: difference Fourier map
Least-squares matrix: full	Hydrogen site location: inferred from neighbouring sites
*R*[*F*^2^ > 2σ(*F*^2^)] = 0.078	H-atom parameters constrained
*wR*(*F*^2^) = 0.154	*w* = 1/[σ^2^(*F*_o_^2^) + (0.*P*)^2^ + 4.3921*P*] where *P* = (*F*_o_^2^ + 2*F*_c_^2^)/3
*S* = 1.06	(Δ/σ)_max_ < 0.001
1991 reflections	Δρ_max_ = 0.42 e Å^−^^3^
155 parameters	Δρ_min_ = −0.36 e Å^−^^3^
1 restraint	Absolute structure: Flack (1983), 897 Friedel pairs
Primary atom site location: structure-invariant direct methods	Flack parameter: 0.3 (2)

### Special details

**Table:**

Experimental. *SADABS* was used to perform the Absorption correction. Parameter refinement on 6249 reflections reduced *R*(int) from 0.1275 to 0.0768. Ratio of minimum to maximum apparent transmission: 0.627938. The given Tmin and Tmax were generated using the *SHELX* SIZE command
Geometry. All s.u.'s (except the s.u. in the dihedral angle between two l.s. planes) are estimated using the full covariance matrix. The cell s.u.'s are taken into account individually in the estimation of s.u.'s in distances, angles and torsion angles; correlations between s.u.'s in cell parameters are only used when they are defined by crystal symmetry. An approximate (isotropic) treatment of cell s.u.'s is used for estimating s.u.'s involving l.s. planes.
Refinement. Refinement of *F*^2^ against ALL reflections. The weighted *R*-factor *wR* and goodness of fit *S* are based on *F*^2^, conventional *R*-factors *R* are based on *F*, with *F* set to zero for negative *F*^2^. The threshold expression of *F*^2^ > 2σ(*F*^2^) is used only for calculating *R*-factors(gt) *etc*. and is not relevant to the choice of reflections for refinement. *R*-factors based on *F*^2^ are statistically about twice as large as those based on *F*, and *R*- factors based on ALL data will be even larger.

### Fractional atomic coordinates and isotropic or equivalent isotropic displacement parameters (Å^2^)

**Table:**

	*x*	*y*	*z*	*U*_iso_*/*U*_eq_	
C1	0.1251 (6)	0.3579 (8)	0.7101 (8)	0.0287 (19)	
C2	0.2293 (5)	0.3958 (7)	0.7510 (9)	0.0232 (17)	
C3	0.2434 (6)	0.4986 (7)	0.8427 (10)	0.037 (2)	
H3	0.3127	0.5226	0.8751	0.045*	
C4	0.1569 (6)	0.5650 (8)	0.8861 (9)	0.037 (2)	
H4	0.1667	0.6362	0.9469	0.044*	
C5	0.0545 (6)	0.5299 (8)	0.8424 (9)	0.037 (2)	
H5	−0.0043	0.5775	0.8740	0.044*	
C6	0.0372 (6)	0.4267 (8)	0.7537 (9)	0.034 (2)	
H6	−0.0326	0.4034	0.7233	0.040*	
C7	0.2709 (5)	0.2345 (7)	0.6117 (11)	0.0262 (18)	
C8	0.4331 (5)	0.1292 (8)	0.5459 (9)	0.030 (2)	
N1	0.3104 (5)	0.3205 (6)	0.6934 (7)	0.0294 (16)	
N2	0.3265 (4)	0.1462 (6)	0.5312 (7)	0.0295 (16)	
H2	0.2908	0.0978	0.4663	0.035*	
N3	0.4938 (5)	0.2037 (6)	0.6268 (7)	0.0331 (17)	
H3A	0.5628	0.1920	0.6311	0.040*	
H3B	0.4655	0.2654	0.6768	0.040*	
N4	0.4741 (5)	0.0366 (6)	0.4707 (8)	0.0347 (17)	
H4A	0.5430	0.0241	0.4743	0.042*	
H4B	0.4326	−0.0128	0.4168	0.042*	
S1	0.13101 (12)	0.22884 (17)	0.5956 (3)	0.0316 (5)	
C11	0.2365 (6)	−0.0922 (8)	0.3103 (10)	0.035 (2)	
C12	0.1654 (6)	−0.1819 (8)	0.2300 (10)	0.039 (2)	
H12A	0.1089	−0.1410	0.1695	0.058*	
H12B	0.1340	−0.2329	0.3149	0.058*	
H12C	0.2069	−0.2304	0.1531	0.058*	
O11	0.3349 (4)	−0.1038 (6)	0.2875 (7)	0.0417 (15)	
O12	0.1938 (4)	−0.0085 (5)	0.3904 (7)	0.0372 (14)	

### Atomic displacement parameters (Å^2^)

**Table:**

	*U*^11^	*U*^22^	*U*^33^	*U*^12^	*U*^13^	*U*^23^
C1	0.026 (4)	0.041 (6)	0.019 (4)	0.002 (4)	−0.003 (3)	0.003 (3)
C2	0.022 (4)	0.019 (4)	0.029 (4)	0.001 (3)	−0.004 (3)	0.000 (3)
C3	0.018 (3)	0.062 (6)	0.032 (4)	−0.006 (5)	0.002 (3)	0.004 (6)
C4	0.038 (5)	0.035 (6)	0.037 (5)	0.000 (4)	0.008 (4)	0.002 (4)
C5	0.026 (4)	0.045 (7)	0.040 (5)	0.002 (4)	0.007 (4)	−0.008 (4)
C6	0.016 (4)	0.049 (6)	0.035 (5)	0.007 (4)	0.003 (3)	0.010 (5)
C7	0.022 (3)	0.036 (5)	0.021 (4)	0.005 (3)	0.003 (4)	0.007 (4)
C8	0.022 (4)	0.037 (5)	0.031 (5)	−0.003 (4)	−0.001 (3)	0.010 (4)
N1	0.017 (3)	0.040 (5)	0.031 (4)	0.002 (3)	0.000 (3)	−0.003 (3)
N2	0.019 (3)	0.040 (5)	0.029 (4)	0.000 (3)	0.001 (3)	−0.002 (3)
N3	0.022 (3)	0.040 (5)	0.037 (4)	0.009 (3)	−0.006 (3)	−0.001 (4)
N4	0.015 (3)	0.037 (5)	0.052 (4)	0.003 (3)	0.001 (3)	0.001 (4)
S1	0.0167 (7)	0.0426 (13)	0.0355 (10)	−0.0010 (9)	−0.0014 (10)	−0.0046 (12)
C11	0.024 (5)	0.045 (6)	0.035 (5)	−0.002 (4)	−0.001 (4)	0.000 (4)
C12	0.029 (4)	0.041 (6)	0.046 (5)	−0.002 (4)	0.003 (4)	−0.006 (4)
O11	0.018 (3)	0.049 (4)	0.058 (4)	0.000 (3)	0.006 (2)	−0.015 (3)
O12	0.021 (3)	0.045 (4)	0.045 (3)	−0.003 (3)	0.002 (3)	−0.010 (3)

### Geometric parameters (Å, °)

**Table:**

C1—C6	1.398 (10)	C8—N3	1.311 (9)
C1—C2	1.420 (10)	C8—N4	1.314 (9)
C1—S1	1.727 (8)	C8—N2	1.361 (8)
C2—C3	1.388 (11)	N2—H2	0.8800
C2—N1	1.408 (9)	N3—H3A	0.8800
C3—C4	1.368 (10)	N3—H3B	0.8800
C3—H3	0.9500	N4—H4A	0.8800
C4—C5	1.395 (11)	N4—H4B	0.8800
C4—H4	0.9500	C11—O11	1.260 (9)
C5—C6	1.384 (11)	C11—O12	1.265 (10)
C5—H5	0.9500	C11—C12	1.499 (10)
C6—H6	0.9500	C12—H12A	0.9800
C7—N1	1.275 (10)	C12—H12B	0.9800
C7—N2	1.381 (10)	C12—H12C	0.9800
C7—S1	1.768 (6)		
			
C6—C1—C2	120.4 (7)	N3—C8—N2	121.9 (8)
C6—C1—S1	129.6 (6)	N4—C8—N2	117.3 (7)
C2—C1—S1	109.8 (6)	C7—N1—C2	110.3 (6)
C3—C2—N1	126.0 (7)	C8—N2—C7	124.1 (7)
C3—C2—C1	119.7 (7)	C8—N2—H2	118.0
N1—C2—C1	114.3 (7)	C7—N2—H2	118.0
C4—C3—C2	119.5 (8)	C8—N3—H3A	120.0
C4—C3—H3	120.3	C8—N3—H3B	120.0
C2—C3—H3	120.3	H3A—N3—H3B	120.0
C3—C4—C5	121.1 (8)	C8—N4—H4A	120.0
C3—C4—H4	119.5	C8—N4—H4B	120.0
C5—C4—H4	119.5	H4A—N4—H4B	120.0
C6—C5—C4	121.1 (8)	C1—S1—C7	88.5 (4)
C6—C5—H5	119.5	O11—C11—O12	124.8 (8)
C4—C5—H5	119.5	O11—C11—C12	117.0 (8)
C5—C6—C1	118.2 (7)	O12—C11—C12	118.2 (7)
C5—C6—H6	120.9	C11—C12—H12A	109.5
C1—C6—H6	120.9	C11—C12—H12B	109.5
N1—C7—N2	126.5 (6)	H12A—C12—H12B	109.5
N1—C7—S1	117.0 (6)	C11—C12—H12C	109.5
N2—C7—S1	116.4 (6)	H12A—C12—H12C	109.5
N3—C8—N4	120.7 (7)	H12B—C12—H12C	109.5

### Hydrogen-bond geometry (Å, °)

**Table:**

*D*—H···*A*	*D*—H	H···*A*	*D*···*A*	*D*—H···*A*
N2—H2···O12	0.88	1.82	2.671 (8)	162.
N3—H3A···O11^i^	0.88	2.06	2.760 (8)	136.
N3—H3B···N1	0.88	2.05	2.713 (9)	131.
N4—H4A···O12^ii^	0.88	2.03	2.861 (7)	158.
N4—H4B···O11	0.88	1.91	2.790 (8)	173.

Symmetry codes: (i) −*x*+1, −*y*, *z*+1/2; (ii) *x*+1/2, −*y*, *z*.
